# Save a trip: Clinical outcomes of cardiac allografts recovered by local surgeons compared to recipient center surgeons.

**DOI:** 10.1016/j.jhlto.2025.100217

**Published:** 2025-01-22

**Authors:** Awab Ahmad, Timothy R. Harris, Aaron Williams, Alexandra DeBose-Scarlett, Rubayet Kamal, Enock Atta Adjei, Hasan K. Siddiqi, Chen Chia Wang, Mark Petrovic, Clifton D. Keck, Shelley R. Scholl, Ashish S. Shah, Swaroop Bommareddi, Brian Lima, John M. Trahanas

**Affiliations:** a Department of Cardiac Surgery, Vanderbilt University Medical Center, Nashville TN; b Meharry Medical College School of Medicine, Nashville TN; c Department of Cardiology, Vanderbilt University Medical Center, Nashville TN

**Keywords:** local organ recovery, heart transplant, primary graft dysfunction, organ, preservation

## Abstract

**Background:**

Local surgeon recovery of donor livers and kidneys is common and well-studied. This practice is rare and poorly studied in cardiac transplantation. We examined clinical outcomes of cardiac allografts recovered by local surgeons vs. recipient institution surgeons.

**Methods:**

A retrospective review of all recoveries of adult cardiac allografts from brain dead donors for a single academic transplant center between 1/2020 and 12/2021 was performed. Donor and recipient baseline characteristics, distance traveled and ischemic time, and recipient outcomes were collected. Primary graft dysfunction (PGD) was determined based on 2014 ISHLT guidelines.

**Results:**

218 recovery attempts were included, 25 conducted by local surgeons. Donor demographics between the two groups were similar, with a mean age of 31.5±10 years. There was a non-significant trend towards higher acceptance rates by local surgeons compared to recipient center surgeons (96% vs 82.9%, p=0.139). Ischemic times (208±28 vs 176±61 min p=0.003) and travel distances (788 vs 615 miles, p=0.011) were longer in the local recovery group. There was no difference in severe PGD (4.2% vs 7.5%, p=1.0), moderate/severe PGD (12.5% vs 14.4%, p=0.22) or 30-day survival (95.8% vs 95%, p=0.218) between the local surgeon and recipient center recovered allografts.

**Conclusion:**

Cardiac allografts recovered by a local surgeon team are high quality with similar rates of organ acceptance, PGD, and 30-day survival. This provides evidence that leveraging the expertise of the local surgical team is a safe and effective method for decreasing travel risks, financial expenditure, and opportunity cost associated with cardiac allograft recovery.

## Graphical abstract

**Figure:**
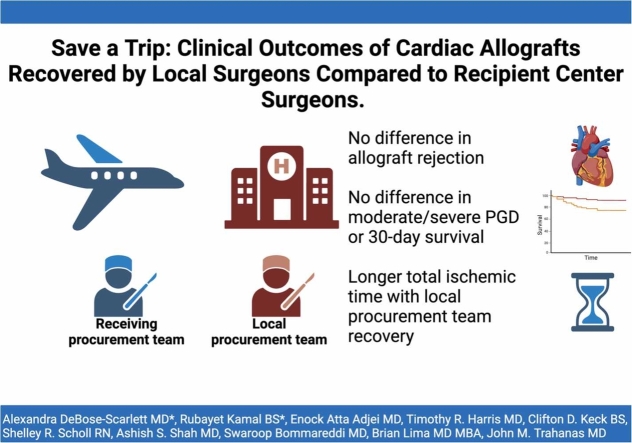

## Introduction

Unlike most surgical careers, a position as a transplant surgeon often includes responsibilities for organ recovery. This elevates levels of occupational hazard due to the urgent nature of the travel involved. In 2012, a cardiac transplant surgeon, technician, and pilot departed by helicopter to recover a cardiac allograft for a patient in need of transplant.^1^ Unfortunately, the helicopter crashed after takeoff and everyone on board perished. Work related fatalities are a small, but real risk for the transplant surgeon.

Highlighting this danger, a 2009 survey described the experiences and concerns of transplant personnel.^2^ 15% of the respondents reported being personally involved in a recovery-related travel accident and 33% of respondents reported accidents at their own transplant center related to organ recovery.^2^ However, self-reported attitudes did not reflect this risk: 87% of the respondents reported feeling “safe”, “usually safe”, or “very safe” on organ recovery travel.^2^ A subsequent follow up survey 10 years later found that as many as 23% of respondents reported involvement in at least one recovery related accident with bodily injury and over half cited “near misses.”^3^

The use of a local surgical team to recover the allograft may help reduce the risks associated with surgeon travel. In abdominal transplantation, this strategy is commonly utilized and studies have shown equivalent outcomes between organs recovered by local surgeons versus institutional teams.^4^, ^5^, ^6^, ^7^, ^8^, ^9^ This practice is less common in thoracic transplantation,^8^ and no studies have examined this practice specifically with regard to cardiac transplantation. At our institution, we support the use of local recovery teams when possible and we sought to determine if clinical outcomes are comparable. We examined rate of organ acceptance and clinical recipient outcomes for transplanted hearts recovered by the local surgical team versus those recovered by the surgical team of the recipient center.

## Materials and Methods

A single center retrospective review was performed of all adult heart recovery attempts for our institution as well as all adult patients who underwent heart transplantation at Vanderbilt University Medical Center (VUMC) between 2020 and 2021. All patients over the age of 18 years for whom a heart recovery was attempted were included. Recipients and organs were excluded if they underwent donation after circulatory death (DCD) protocol, as this is a more complex recovery procedure requiring specialized expertise. This study was approved by the Institutional Review Board (#181379) and consent was determined to not be required for this retrospective chart review. This study complies with the ISHLT Ethics statement.

Demographic data and baseline clinical information for both the donor and recipient were gathered. The surgical team that recovered the donor allograft was identified as either from VUMC or as a local recovery surgeon/team. The decision to use a local recovery team was primarily based on logistical considerations. If our retrieval team was engaged in another recovery or fatigued from multiple recoveries during the week, and a local team was available, the local team was selected to perform the recovery. Round trip distance was calculated for each organ recovery.

Whether or not the potential donor organ was ultimately utilized or rejected was noted. Ischemic time was taken as the total ischemic time. Postoperative outcomes were analyzed. New post-operative extracorporeal membrane oxygenation (ECMO) requirement was recorded. Our center used the University of Wisconsin (UW) solution for allograft preservation until January 21, 2021, after which we transitioned to Del Nido solution. However, due to the lack of available information on the preservation solutions used by local surgeons, we did not include this factor in our analysis. Primary graft dysfunction (PGD) at 24 h after transplant was determined based on ISHLT guidelines.^10^ 30-day mortality was also reviewed.

Descriptive statistics were calculated for demographic data and baseline characteristics. Student’s t tests and Wilcoxon rank sum tests were utilized, as appropriate, to compare the donors and recipients whose allografts were recovered by VUMC vs local surgeons. Categorical variables were compared using Chi-squared and Fisher’s exact tests. Two-sided tests and an alpha level of 0.05 were taken for all analyses. Statistical analysis was conducted in R 4.3.0 (R Core Team).

## Results

218 potential cardiac allografts were included in the study, 25 (11.5%) of which were evaluated by local surgeons and 193(88.5%) by VUMC surgeons **(**Table 1**)**. The average age of the donors was 31 years in the local surgeon group and 32 years for VUMC surgeons (p=0.862). 32% were female in both groups, and the average BMI (body mass index) was 25 for local surgeons and 27 for VUMC surgeons (p=0.24). Cause of death was statistically different between groups, whereas procurements performed by local surgeons had lower rates of donations due to anoxia (40% vs 58%), but higher rates of donations secondary to trauma (40% vs 24%, p=0.04). Cardiopulmonary resuscitation (CPR) was performed on 60% of local surgeon allografts and 66% of VUMC allografts (p=0.38), with no difference in CPR time (27 vs. 24 min, p=0.39). 12% of local surgeon hearts had prior HCV (hepatitis C virus) exposure while 22% were exposed to HCV for the VUMC surgeons (p=0.31). However, ischemic time was significantly longer with local surgeons having an average ischemic time of 208 min while VUMC surgeons averaged 176 min (p=0.003). Trip distance was similarly longer for local surgeons (788 nautical miles) than VUMC surgeons (615 nautical miles, p=0.01). A quantile regression analysis, which, after adjusting for donor distance (i.e., one-way trip from donor to recipient center), showed that the difference in ischemic times between the two teams was no longer significant (p = 0.67). Additionally, donor distance was categorized into four intervals: 0–200, 200–400, 400–600, and >600 nautical miles. In all distance categories, the ischemic time differences between the two teams remained non-significant. (p value > 0.1 in all categories). 96% of allografts recovered by local surgeons were ultimately implanted, while 83% of those recovered by VUMC surgeons were ultimately implanted into recipients (p=0.14).

**Table 1:** Donor Characteristics.

	**All**	**Local Surgeon**	**VUMC Surgeon**	**P-Value**
	N= 218	N= 25 (11.5%)	N= 193 (88.5%)	
**Age (years)**	31.5 (10.1)	31.2 (11.1)	31.5 (10)	0.8621
**Distance+**	632.8 (477.2)	788 (303)	615 (491)	0.0113*
**BMI (kg/m**^**2**^**)**	27 (6.7)	24.9 (4.6)	27.3 (6.9)	0.2379
**CPR (minutes)**	23.8 (17.6)	26.9 (16.9)	23.5 (17.7)	0.3856
**LVEF (%)**	60 (7.2)	58.6 (6)	60.1 (7.4)	0.2774
**Ischemic Time (minutes)**^**+**^	179.5 (59.3)	208 (28.4)	176 (61.2)	0.0033*
**Sex**				1.0
Female	69 (31.7%)	8 (32%)	61 (31.6%)	
Male	149 (68.3%)	17 (68%)	132 (68.4%)	
**Race**				0.1269
American Native	3 (1.4%)	0 (0%)	3 (1.6%)	
Asian	3 (1.4%)	1 (4%)	2 (1%)	
Black	34 (15.6%)	6 (24%)	28 (14.5%)	
Hispanic	24 (11%)	0 (0%)	24 (12.4%)	
White	154 (70.6%)	18 (72%)	136 (70.5%)	
**Cause of Death**				0.0414*
Anoxia	122 (56%)	10 (40%)	112 (58%)	
CNS Tumor	1 (0.5%)	1 (4%)	0 (0%)	
Other	11 (5.1%)	0 (0%)	11 (5.7%)	
Stroke	26 (11.9%)	4 (16%)	22 (11.4%)	
Trauma	58 (26.6%)	10 (40%)	48 (24.9%)	
**CPR Administered**				0.3841
No	72 (33%)	9 (36%)	63 (32.6%)	
Unknown	4 (1.8%)	1 (4%)	3 (1.6%)	
Yes	142 (65.1%)	15 (60%)	127 (65.8%)	
**Anti HCV ab**				0.3067
No	173 (79.4%)	22 (88%)	151 (78.2%)	
Yes	45 (20.6%)	3 (12%)	42 (21.8%)	
**Organ Decision**				0.1390
Accepted	184 (84.4%)	24 (96%)	160 (82.9%)	
Declined	34 (15.6%)	1 (4%)	33 (17.1%)	

BMI= Body Mass Index, CNS= Central Nervous System, CPR= Cardiopulmonary Resuscitation, HCV= Hepatitis C Virus, LVEF= Left Ventricular Ejection Fraction, VUMC= Vanderbilt University Medical Center

184 heart transplants were performed, with 24 (13%) of the allografts recovered by local surgeons and 160 (87%) by VUMC surgeons **(**Table 2**)**. The average age of transplanted hearts was 32 years in both groups (p=0.836) with similar proportions of female donors in the local and VUMC surgeon groups (33% vs. 35%, p=1.000). Donor BMI (25.3 vs 27.5, p=0.345) and left ventricular ejection fraction (LVEF) (59% vs 60%, p= 0.252) were also similar between local and VUMC surgeons. Trip distance between the recipient and donor institutions was greater for allografts recovered by the local surgeons (788 nautical miles) compared to the VUMC surgeons (600 nautical miles, p=0.007).

**Table 2:** Transplanted Donor Characteristics.

	**All**	**Local Surgeon**	**VUMC Surgeon**	**P-Value**
	N= 184	N= 24 (13.0%)	N= 160 (87.0%)	
**Age (years)**	31.9 (10)	31.5 (11.3)	31.9 (9.9)	0.8355
**Distance**	623 (463.2)	788 (303)	600 (477)	0.00697*
**BMI (kg/m**^**2**^**)**	27.2 (6.8)	25.3 (4.2)	27.5 (7)	0.3448
**CPR (minutes)**	25.2 (18)	26.9 (16.9)	25 (18.2)	0.5805
**LVEF (%)**	60.2 (7.4)	58.8 (6.1)	60.4 (7.6)	0.2524
**Ischemic Time (minutes)**^**+**^	179.5 (59.3)	208.0 (28.4)	176 (61.2)	0.00332*
**Sex**				1.0000
Female	64 (34.8%)	8 (33.3%)	56 (35%)	
Male	120 (65.2%)	16 (66.7%)	104 (65%)	
**Race**				0.1461
American Native	3 (1.6%)	0 (0%)	3 (1.9%)	
Asian	3 (1.6%)	1 (4.2%)	2 (1.3%)	
Black	30 (16.3%)	6 (25%)	24 (15%)	
Hispanic	20 (10.9%)	0 (0%)	20 (12.5%)	
White	128 (69.6%)	17 (70.8%)	111 (69.4%)	
**Cause of Death**				0.05452
Anoxia	104 (56.5%)	10 (41.7%)	94 (58.8%)	
CNS Tumor	1 (0.5%)	1 (4.2%)	0 (0%)	
Other	10 (5.4%)	0 (0%)	10 (6.3%)	
Stroke	22 (12%)	4 (16.7%)	18 (11.2%)	
Trauma	47 (25.5%)	9 (37.5%)	38 (23.8%)	
**CPR Administered**				0.4454
No	61 (33.2%)	9 (37.5%)	52 (32.5%)	
Unknown	4 (2.2%)	1 (4.2%)	3 (1.9%)	
Yes	119 (64.7%)	14 (58.3%)	105 (65.6%)	
**Anti HCV ab**				0.2964
No	142 (77.2%)	21 (87.5%)	121 (75.6%)	
Yes	42 (22.8%)	3 (12.5%)	39 (24.4%)	

BMI= Body Mass Index, CNS= Central Nervous System, CPR= Cardiopulmonary Resuscitation, HCV= Hepatitis C Virus, LVEF= Left Ventricular Ejection Fraction, VUMC= Vanderbilt University Medical Center

Heart transplant recipient profile was similar between the local surgeon and VUMC surgeon cohorts **(**Table 3**)**. The average age was 55 years in both groups (p= 0.975). 11 (46%) recipients were female in the local surgeon group, while 28% were female in the VUMC surgeon group, which did not rise to the level of significance (p=0.0503). 18 (75%) and 111 (69%) recipients identified as “white/Caucasian”, respectively (p=0.557). Approximately half of recipients in the local vs. VUMC groups, respectively, had a prior diagnosis of coronary artery disease (13 (54%) vs 78 (49%), p=0.206) as well as chronic kidney disease (13 (54%) vs 91 (57%), p=0.231). Diabetes mellitus was present in 21% of the local surgeon cohort and 36% in the VUMC surgeon cohort (p=0.090). Chronic obstructive pulmonary disease was present in 4 (17%) and 19 (12%) in the local surgeon and VUMC surgeon groups (p=0.172). Lastly, cirrhosis was rare, with only 1 individual (4%) diagnosed in the local surgeon group and 14 (9%) in the VUMC surgeon group (p=0.185).

**Table 3:** Recipient Characteristics.

	**All**	**Local Surgeon**	**VUMC Surgeon**	**P-Value**
	N= 184	N= 24 (13%)	N= 160 (87%)	
**Age (years)**	54.6 (11.5)	54.8 (11)	54.6 (11.6)	0.9754
**Sex**				0.0503
Female	56 (30.4%)	11 (45.8%)	45 (28.1%)	
Male	128 (69.6%)	13 (54.2%)	115 (71.9%)	
**Race**				0.5569
Asian	2 (1.1%)	0 (0%)	2 (1.3%)	
Black	49 (26.6%)	5 (20.8%)	44 (27.5%)	
Hispanic	3 (1.6%)	1 (4.2%)	2 (1.3%)	
Other	1 (0.5%)	0 (0%)	1 (0.6%)	
White	129 (70.1%)	18 (75%)	111 (69.4%)	
**Coronary Artery Disease**				0.2057
Yes	91 (49.5%)	13 (54.2%)	78 (48.8%)	
**Chronic Obstructive Pulmonary Disease**				0.1716
Yes	23 (12.5%)	4 (16.7%)	19 (11.9%)	
**Chronic Kidney Disease**				0.2312
Yes	104 (56.5%)	13 (54.2%)	91 (56.9%)	
**Diabetes Mellitus**				0.08974
Yes	62 (33.7%)	5 (20.8%)	57 (35.6%)	
**Cirrhosis**				0.1847
Yes	15 (8.2%)	1 (4.2%)	14 (8.8%)	
**Obstructive Sleep Apnea**				0.1156
Yes	47 (25.5%)	4 (16.7%)	43 (26.9%)	
**Cerebrovascular Disease**				0.0982
Yes	29 (15.8%)	6 (25%)	23 (14.4%)	
**Cancer History**				0.2039
Yes	20 (10.9%)	3 (12.5%)	17 (10.6%)	
**Peripheral Vascular Disease**				0.2378
Yes	34 (18.5%)	5 (20.8%)	29 (18.1%)	
**Dyslipidemia**				0.1743
Yes	75 (40.8%)	8 (33.3%)	67 (41.9%)	

Notably, recipient outcomes were similar between the hearts recovered by local surgeons and those recovered by VUMC surgeons **(**Table 4**)**. Moderate or severe primary graft dysfunction occurred in 3 patients (13%) of the local surgeon group and 23 patients (14%) of the VUMC surgeon group (p=0.224). Baseline characteristics such as donor and recipient age, sex, BMI, diabetes, hypertension, donor left ventricular ejection fraction, use of inotropes, history of cardiac arrest or CPR, predicted heart mass ratio, female-to-male mismatch, recipient history of sternotomy or retransplant, preoperative creatinine levels, preoperative LVAD or ECMO support, pulmonary vascular resistance, heart failure cause, waitlist status, multiorgan transplant, and total ischemic times were all considered for the multivariable regression model. Variables were selected by adding and removing them based on a significance level of p < 0.1. The final model included multiorgan transplant, recipient BMI, and history of sternotomy. After adjusting for these factors, procurement by the local team did not significantly impact the incidence of PGD (p = 0.19). Extracorporeal membrane oxygenation (ECMO) utilization within the first 24 h was rare, with 1 (4%) of the local surgeon group and 12 (8%) of the VUMC surgeon group needing ECMO (p=1.000). Finally, 30-day mortality was the same in both groups at 4.2% and 5% (p=0.218).

**Table 4:** Recipient Outcomes.

	**All**	**Local Surgeon**	**VUMC Surgeon**	**P-Value**
	N= 184	N= 24 (13%)	N= 160 (87%)	
**Required ECMO**				1.0
No	171 (92.9%)	23 (95.8%)	148 (92.5%)	
Yes	13 (7.1%)	1 (4.2%)	12 (7.5%)	
**PGD**				0.2240
No	158 (85.9%)	21 (87.5%)	137 (85.6%)	
Yes	26 (14.1%)	3 (12.5%)	23 (14.4%)	
**30 Day Mortality**				0.2182
No	175 (95.1%)	23 (95.8%)	152 (95%)	
Yes	9 (4.9%)	1 (4.2%)	8 (5%)	

ECMO= Extracorporeal Membrane Oxygenation, PGD= Primary Graft Dysfunction

Secondary analysis was undertaken to examine the final decision for hearts that were recovered by each team, whether the allograft was implanted into a recipient or declined **(**Table 5**)**. Of the 215 heart allografts evaluated, 184 hearts were implanted (24 by local surgeons and 193 by VUMC surgeons) while 34 were declined (1 and 33, respectively, p=0.139). The profile of accepted vs declined allografts was overall similar between local and VUMC surgeon groups, with the average age at 32 vs 30 years (p=0.243), average BMI of 27 kg/m^2^ vs 26 kg/m^2^ (p=0.501), and average LVEF of 60% vs 59% (p=0.151). Average trip distance was nearly identical at 623 nautical miles for accepted hearts vs 692 nautical miles for declined hearts (p=0.643). Hearts from female donors tended to be accepted more than hearts from male donors with an acceptance rate of 93% vs 81% (p=0.035). The use of CPR was also similar at 65% of accepted hearts having received CPR and 68% of declined hearts receiving CPR (p=1.000), however, duration of CPR was longer in the accepted hearts at 25 min vs 16 min (p=0.018). The cause of death profile, however, was similar with anoxia being the most common at 57% vs 53% and trauma at 26% vs 32% for accepted vs declined hearts respectively (p=0.860). Hisotry of HCVexposure was 23% in the accepted allografts and 9% in the declined hearts, however, this did not meet the threshold of statistical significance (p=0.068).

**Table 5:** Donor Characteristics of Accepted versus Declined Allografts.

	**All**	**Accepted**	**Declined**	**P-Value**
	N= 218	N= 184 (84.4%)	N= 34 (15.6%)	
**Age (years)**	31.5 (10.1)	31.9 (10)	29.5 (10.1)	0.2427
**Miles Traveled**	632.8 (477.2)	623 (463)	692 (560)	0.6425
**BMI (kg/m**^**2**^**)**	27 (6.7)	27.2 (6.8)	26.2 (6.2)	0.5014
**CPR (minutes)**	23.8 (17.6)	25.2 (18)	15.7 (12.8)	0.0184*
**LVEF (%)**	60 (7.2)	60.2 (7.4)	59 (6)	0.1507
**Ischemic Time (minutes)**^**+**^	179.5 (59.3)	179 (59.3)	n/a	n/a
**Sex**				0.0347*
Female	69 (31.7%)	64 (34.8%)	5 (14.7%)	
Male	149 (68.3%)	120 (65.2%)	29 (85.3%)	
**Race**				0.9376
American Native	3 (1.4%)	3 (1.6%)	0 (0%)	
Asian	3 (1.4%)	3 (1.6%)	0 (0%)	
Black	34 (15.6%)	30 (16.3%)	4 (11.8%)	
Hispanic	24 (11%)	20 (10.9%)	4 (11.8%)	
White	154 (70.6%)	128 (69.6%)	26 (76.5%)	
**Cause of Death**				0.8596
Anoxia	122 (56%)	104 (56.5%)	18 (52.9%)	
CNS Tumor	1 (0.5%)	1 (0.5%)	0 (0%)	
Other	11 (5.1%)	10 (5.4%)	1 (2.9%)	
Stroke	26 (11.9%)	22 (12%)	4 (11.8%)	
Trauma	58 (26.6%)	47 (25.5%)	11 (32.4%)	
**CPR Administered**				1.0
No	72 (33%)	61 (33.2%)	11 (32.4%)	
Unknown	4 (1.8%)	4 (2.2%)	0 (0%)	
Yes	142 (65.1%)	119 (64.7%)	23 (67.6%)	
**Anti HCV ab**				0.0683
No	173 (79.4%)	142 (77.2%)	31 (91.2%)	
Yes	45 (20.6%)	42 (22.8%)	3 (8.8%)	
**Surgeon**				0.1390
Local Surgeon	25 (11.5%)	24 (13%)	1 (2.9%)	
VUMC Surgeon	193 (88.5%)	160 (87%)	33 (97.1%)	

BMI= Body Mass Index, CNS= Central Nervous System, CPR= Cardiopulmonary Resuscitation, HCV= Hepatitis C Virus, LVEF= Left Ventricular Ejection Fraction, VUMC= Vanderbilt University Medical Center

## Discussion

In our experience with donation after brain death (DBD) cardiac transplantation, we found that cardiac allografts recovered by a local surgical team were similar to hearts recovered by our own VUMC team. Hearts evaluated by local surgeons during procurement were accepted at similar rates as hearts evaluated by our recipient institutional team. As expected, hearts recovered by local surgeons were further away leading to longer ischemic times. However, short term outcomes, including the need for ECMO and moderate/severe PGD within 24 h, were similar between both groups. Interestingly, hearts from female donors tended to be accepted more frequently, possibly due to better size matching with smaller recipients. This study provides early evidence that using local surgeons to recover cardiac allografts is a reasonable alternative to sending the recipient team.

Reducing recipient center surgeon travel is important to minimize travel related accidents. Schenk et al. highlight strategies to lower travel risks, including using nonrecipient surgeon groups for organ recovery outside institutions.^3^ Developing dedicated recovery centers may become instrumental in decreasing travel risk. Additionally, standardized accident reporting is recommended to accurately assess and mitigate risks.^3^

In 2014, Doyle et al. described their experience transitioning from transplant team recovery to utilizing a dedicated Organ Procurement Organization (OPO) facility for DBD liver evaluation and recovery in Missouri.^11^ The rate of organ recovery was the same as traditional in-hospital recovery; similarly there was no difference in graft failure and recipient survival at 1, 5, and 10 years.^11^ Unsurprisingly, cold ischemic times and surgical team distance traveled time were decreased.^11^ They found that utilizing a dedicated facility decreased costs by 37%, due to strategies such as absence of premium hospital charges, earlier workup of organ staging by OPO personnel, and removal of transportation costs.

Since not every facility has the resources or transplant volume to develop a dedicated organ recovery center, utilizing local surgeon recovery is another viable strategy to decrease risk and resource utilization. Studies in the abdominal transplant literature have examined the use of local surgeon organ recovery. An experience for liver transplantation in Brazil was described in a 2012 paper by Salvalaggio et al.^6^ Their results are notable in that there was no difference in post-transplant mortality and that there was a trend toward locally recovered organs having improved survival, however this was not statistically significant.^6^ Another study examining liver, kidney, and pancreas transplantation similarly found that there was no difference in outcomes when the organs were recovered by other recovery teams.^7^ This multicenter, multiorgan study examined both short term outcomes and longer-term survival. For each organ, cold ischemic times were longer for the local surgeon team.^7^ Ultimately, however, the organs demonstrated similar rates of death-censored graft failure and patient mortality at 1 and 5 years.^7^ Our study complements this data and similarly demonstrates comparable outcomes for cardiac allografts in DBD donors.

While we excluded DCD recoveries as we did not use local recovery teams for these donors owing to complexities involved with technique, the use of local recovery in DCD donors has been examined in the abdominal transplant literature by Jadloweic et al.^12^ They have shown similar postoperative and long term survival outcomes, despite significant variation in surgical techniques among recovery teams. Future studies should examine outcomes comparing the utilization of local vs. recipient team surgeons for DCD heart recoveries, which are often more technically challenging and involve complex perfusion systems. This is particularly relevant in the current era as commercially available organ recovery services have increased in popularity and are offering DCD recovery.

## Limitations

This is a retrospective study and carries the inherent limitation of possible unidentified confounders. There was a selection bias in choosing the local surgeon teams based on distance and workload. The population size between comparison groups was imbalanced especially lower percentage of recoveries in the local surgeon group which may affect the power and precision of study. Despite these limitations, this study provides some evidence that the use of local recovery teams, with clear organ acceptance criteria, may be a reasonable option for cardiac allograft recovery.

While not statistically significant, there was a trend towards a difference in the ultimate decision between local and VUMC recovery teams. In our practice, senior fellows typically perform allograft recoveries for our transplant recipients, whereas local recovery teams are composed of attending surgeons. One could hypothesize that the higher rejection rate of VUMC-recovered allografts may be attributed to the relative inexperience of the fellows compared to the more seasoned local attending surgeons, who may be better equipped to evaluate allografts. It is possible that the less experienced fellows might unintentionally cause graft injuries during recovery, rendering the allograft unsuitable for transplantation. However, we currently lack detailed data to statistically assess this potential issue. Another possibility is selection bias in the decision to send a VUMC team when there were preexisting concerns about a particular donor or allograft. For instance, if certain donor or allograft characteristics made them less ideal for our recipient, we may have opted to send our own team to further evaluate the organ. That said, we believe this scenario is less likely, as the major characteristics of donors were largely comparable between the VUMC and local recovery teams.

## Conclusions

Our study finds that, similar to what has been observed for abdominal organs, the utilization of a local recovery team to recover cardiac allografts has no impact on the likelihood of accepting the organ, as well as outcomes including the post-operative ECMO, the incidence of PGD, and early mortality. The use of local recovery teams may be a safe option for reducing transplant team risk related to travel as well as recovery costs.

## Financial Conflict of Interest Statement

The authors declare that they have no financial conflicts of interest.

## Author Contributions

ADS- substantial contributions to study conception, data acquisition, data analysis, data interpretation, writing, and editing. RK- substantial contributions to study conception, data acquisition, data interpretation, writing, and editing. EAA- substantial contributions to study conception, data acquisition, data interpretation, writing and editing. TRH- substantial contributions to editing, formatting, and submission. CDK- substantial contributions to data acquisition and editing. SRS- substantial contributions to study conception, data acquisition, and editing. ASS- substantial contributions to study conception and editing. SB- substantial contributions to editing. CP- substantial contributions to data acquisition, data analysis, data interpretation, and editing. JT- substantial contributions to study conception, data acquisition, data analysis, data interpretation, writing, and editing.
